# DSIF modulates RNA polymerase II occupancy according to template G + C content

**DOI:** 10.1093/nargab/lqac054

**Published:** 2022-07-27

**Authors:** Ning Deng, Yue Zhang, Zhihai Ma, Richard Lin, Tzu-Hao Cheng, Hua Tang, Michael P Snyder, Stanley N Cohen

**Affiliations:** Department of Genetics, Stanford University School of Medicine, Stanford, CA 94305, USA; Department of Genetics, Stanford University School of Medicine, Stanford, CA 94305, USA; Department of Genetics, Stanford University School of Medicine, Stanford, CA 94305, USA; Department of Genetics, Stanford University School of Medicine, Stanford, CA 94305, USA; Institute of Biochemistry and Molecular Biology, National Yang Ming Chiao Tung University, Taipei 112, Taiwan; Department of Genetics, Stanford University School of Medicine, Stanford, CA 94305, USA; Department of Genetics, Stanford University School of Medicine, Stanford, CA 94305, USA; Department of Genetics, Stanford University School of Medicine, Stanford, CA 94305, USA

## Abstract

The DSIF complex comprising the Supt4h and Supt5h transcription elongation proteins clamps RNA polymerase II (RNAPII) onto DNA templates, facilitating polymerase processivity. Lowering DSIF components can differentially decrease expression of alleles containing nucleotide repeat expansions, suggesting that RNAPII transit through repeat expansions is dependent on DSIF functions. To globally identify sequence features that affect dependence of the polymerase on DSIF in human cells, we used ultra-deep ChIP-seq analysis and RNA-seq to investigate and quantify the genome-wide effects of Supt4h loss on template occupancy and transcript production. Our results indicate that RNAPII dependence on Supt4h varies according to G + C content. Effects of DSIF knockdown were prominent during transcription of sequences high in G + C but minimal for sequences low in G + C and were particularly evident for G + C-rich segments of long genes. Reanalysis of previously published ChIP-seq data obtained from mouse cells showed similar effects of template G + C composition on Supt5h actions. Our evidence that DSIF dependency varies globally in different template regions according to template sequence composition suggests that G + C content may have a role in the selectivity of Supt4h knockdown and Supt5h knockdown during transcription of gene alleles containing expansions of G + C-rich repeats.

## INTRODUCTION

The ability of RNA polymerase II (RNAPII) to initiate transcription of eukaryotic genes and to remain attached to the DNA template during transcript elongation is a highly regulated process (1). Elongation of transcripts is dependent in part on a protein complex called DSIF (5,6-dichloro-1-β-d-ribofuranosylbenzimidazole sensitivity-inducing factor (2,3)), which is formed in mammalian cells by interaction of the Supt4h and Supt5h proteins (4–6). The DSIF complex tightly associates with the phosphorylated carboxy terminal domain of the transcript-elongating form of RNAPII (RNAPII-S2) (7–11) to close a structural cleft in the RNAPII active center. Such closure has been proposed to clamp the multicomponent RNAPII complex onto DNA, reducing RNAPII dissociation from the template and imparting processivity to the polymerase (12,13).

Supt4h and Supt5h and their orthologs have been shown to have multiple biological and biochemical roles beyond the clamping of RNAPII onto DNA templates during transcription elongation, including events that occur during initiation (14) or termination (15) of transcription. Reduction of the Supt4h or Supt5h concentration by an amount that only marginally alters overall transcript production can prominently affect the ability of RNAPII to proceed through DNA regions containing expanded nucleotide repeats, and dependence on Supt4h or Supt5h for efficient transcript production has been observed for mutated alleles of the *HTT* gene (16–19), the orf72 locus on human chromosome 9 (i.e. *C9orf72* (20,21)) and the *NOP56* gene (22)—which are associated respectively with Huntington's Disease, amyotrophic lateral sclerosis and frontotemporal dementia, and SCA36 type ataxia. To better understand the role of template sequence in determining RNAPII reliance on DSIF, we globally investigated and quantified the effects of Supt4h or Supt5h reduction on template occupancy and transcript production by the polymerase.

## MATERIALS AND METHODS

### Supt4h reduction in iPSCs and NPCs

A lentivirus construct carrying human Supt4h shRNA (clone ID: TRCN0000019645 from Dharmacon having the antisense sequence TTAAAGTTACTGACTCGCTGC) and a gene encoding resistance to the antibiotic puromycin was introduced into the iPSC cell line GM23225 (Coriell Institute). Puromycin was used to select cells that acquired the construct. Knock-down of Supt4h in iPSCs and NPCs was confirmed by western blotting (Supplementary Figure S1A). Knock-down of Supt4h in neural progenitor cells (NPCs) for mRNA was additionally confirmed by RNA-seq analysis (Supplementary Table S2).

### iPSC differentiation to NPCs

As described (23), iPSCs were disaggregated from feeder-free cultures using Accutase (Innovative Cell Technologies), and seeded on Matrigel (Corning)-coated plates or on cover slips for staining. The cells were induced to differentiate into neuron progenitor cells in KSR medium (23) comprised of 15% KnockOut Serum Replacement (ThermoFisher Scientific), 1% GlutaMAX, 1% Non-Essential Amino Acids Solution, 1% Gibco™ 2-Mercaptoethanol, 10 μM SB431542 and 500 ng/ml Noggin in KnockOut DMEM (ThermoFisher Scientific). Medium was changed daily for 7 days. Differentiated cells were stained with Pax6 antibody (Stemgent) and Oct4 antibody (Abcam), or collected for RNA-seq and ChIP-seq.

### RNA-seq

Total RNA was extracted using RNeasy kit (Qiagen) according to the manufacturer's instructions. ScriptSeq v2 RNA-seq kit (Epicenter) was used following the manufacturer's instructions. The workflow included rRNA removal (RiboZero technology) followed by RNA fragmentation and ligation-free cDNA synthesis for preparing directional RNA-seq libraries. The amplified libraries were further purified by gel excision and extraction and were analyzed using an Illumina HiSeq 2000 sequencer.

### ChIP-seq analysis

DNA occupancy by RNAPII-S2 was evaluated by ChIP-seq analysis. Human ChIP-seq samples (Supplementary Table S1) produced an average of 378 million 101 bp long paired-end reads. Fastqc (version 0.11.4) was used for assessment of sequence quality. Skewer version 0.2.1 was used to trim adaptor sequences. Reads after adaptor trimming were then aligned to the human (hg19) genome using BWA version 0.7.10. An average of 95% of the reads shared alignment with the reference genome. The transcription start site (TSS) and transcription termination site (TTS) for each gene at the isoform level were defined by RefFlat for hg19 (downloaded from UCSC). Accumulated reads for each gene were assigned according to the most abundant isoform defined in the corresponding untreated RNA-seq sample from GM23225 NPC, as determined by FeatureCount (24,25) version 1.4.6. Read coverage change for each gene was further calculated by DEseq2 version 1.10.1 for these two conditions (UNT and Supt4h-KD) in GM23225 NPC cell lines. Previously published data for RNAPII-S2 ChIP-seq analysis in mouse cells (GEO accession number: GSE106313) were similarly analyzed but mapped instead to the murine genome (mm9).

The procedure used was as previously described (26) with minor modifications. 2 × 10^7^ cells were collected and incubated in 1% formaldehyde for 10 min at room temperature and then quenched by addition of 125mM Glycine. Nuclear lysates were prepared by sonication using a Branson 250 Sonifier. Clarified lysates were treated overnight at 4°C with 5 μg anti-pol II CTD repeat YSPTSPS (phosphor S2) (Abcam ab5095) antibody. Protein–DNA complexes were captured on Protein A/G agarose beads (EMD Millipore) and eluted in 1% SDS TE buffer at 65°C. Following cross-link reversal and purification, ChIP DNA sequencing libraries were prepared according to Illumina DNA sample kit instructions (Illumina). Libraries were sequenced on an Illumina HiSeq 4000 instrument. The experiments were done in two biological replicates (Supplementary Figure S7). More than 300 million reads 101 bp in length were obtained from each library in order to facilitate accurate quantification of RNAPII-S2 occupancy.

### Bioinformatics methods

An average of 59 million 101 bp long paired-end reads was obtained from each human RNA-seq sample (Supplementary Table S2). Fastqc (version 0.11.4) was used for assessment of sequencing quality. Reads were aligned to the Human (hg19) genome using STAR (27) version 2.5.1b and splice junctions were defined in a GTF file (obtained from U.C. Santa Cruz (UCSC) for hg19). An average of 80% of reads were found to align with sequences in the reference genome. Gene expression was determined by calculating reads per kilobase per million aligned reads (FPKM) as well as raw count using RSEM (28) (version 1.2.30). Changes in gene expression associated with knockdown of Supt4h in GM23225 NPC were quantified as fold change (UNT and Supt4h-KD) and calculated by DEseq2 (29) (version 1.10.1).

Mouse RNA-seq (GEO accession number: GSE33497) samples (Supplementary Table S3) had an average of 88 million 101 bp long paired-end reads. Fastqc (version 0.11.4) was used for assessment of sequencing quality. Reads were then aligned to the mouse (mm9) transcriptome with gene annotation being defined in a GTF file (obtained from UCSC for mm9). An average of 75% of reads aligned to the reference sequence. Gene expression was determined by calculating reads per kilobase per million aligned reads (FPKM) as well as by raw count using RSEM (version 1.2.30). Gene expression changes were further calculated using DEseq2 version 1.10.1 for these two conditions (UNT and Supt4h-KD). *P*-value was calculated using Student's *t* test: two-sample assuming equal variances two tails. The Pearson correlation coefficient (*r*) was calculated by PEARSON function of Microsoft Excel to reflect the extent of a linear relationship between data sets.

Gene lengths were defined as the number of nucleotides from the TSS to TTS, including introns. To increase precision in the identification of short template segments occupied by RNAPII-S2, >300 million reads, each ∼100 bp in length, were sequenced for each sample (Supplementary Table S1), producing a total read count that is about 10-fold greater than the number normally obtained in ChIP-seq analysis (30).

### Determination of G + C content

For each gene, we determined the G + C content in the most abundant isoform as defined using a corresponding RNA-seq control sample. A 100 bp sliding window was used to scan the gene body, including exon and intron, from TSS to TTS. We calculated the average G + C% for each sliding window along the gene for heatmap visualization. We also calculated the average G + C% for the whole gene body, excluding the first 1kb after TSS, which for virtually all genes contained a promoter region high in G + C, to represent overall G + C content of the gene.

### Determination of ESKOR

RNAPII-S2 ChIP-seq fragments per kilobase of transcript per million fragments mapped (FPKM) was calculated from the ChIP-seq read count and normalized according to whole gene body length, using procedures employed for RNA-seq FPKM calculations. Genes were chosen for further analysis based on the following criteria: (i) a gene body length (from TSS to TTS including introns) no longer than 5 kb; (ii) RNAPII-S2 ChIP-seq FPKM measurements >0.5 and (iii) transcription of these genes to an extent that resulted in their inclusion also in the RNA-seq list. ESKOR (Effect of Supt4h Knockdown on Occupancy by RNA polymerase) was calculated as log_2_(ChIP-seq_FPKM_Supt4h KD/ChIP-seq_FPKM_UNT). ESKOR value can be either positive or negative. Zero ESKOR represents no difference. Positive ESKOR means more RNAPII occupancy in Supt4_KD than untreated, and vice versa.

## RESULTS

### G + C content of template affects DNA occupancy by RNAPII-S2

Earlier work indicates that knockdown of components of the DSIF complex differentially reduces expression in neural cells of mutant genes containing expanded nucleotide repeats (16–18,21,22). In the experiments reported here, neuron progenitor cells (NPCs) were produced by differentiation of induced human pluripotent stem cells (iPSCs) (23). At the neuron progenitor stage of differentiation, the cells express certain characteristics of neurons but retain the capacity to reproduce in culture (23).

Approximately 30% knockdown of Supt4h in the iPSCs used to generate NPCs was achieved using transfection with shRNA (Materials and Methods) and confirmed by Western blotting (Supplementary Figure S1A). Previous studies have shown that phosphorylation of Serine-2 residues on the RNAPII C-terminal region (CTR) on Supt5 effectively triggers elongation of nascent transcripts (31,32). Staining of *Drosophila* polytene chromosomes indicates that Spt5 co-localizes with the elongating Ser2-phosphorylated form of RNAPII (i.e. RNAPII-S2 (7,8)). We thus used antibody against RNAPII-S2 (Abcam, ab5095) (33) during ChIP-seq analysis, to investigate the effects of DSIF knockdown on DNA occupancy by the polymerase. Occupancy of the template by RNAPII-S2 results in protection of, and therefore read counts for, specific genome sequences (34). Read counts for protected sequences were quantified by their frequency of identification relative to total read counts (i.e. fragments per kilobase of transcript per million assignable mapped reads; FPKM), as normalized for gene length (35). 8779 genes having FPKM values >0.5 were included in our analysis, as indicated in Materials and Methods.

**Figure 1.**
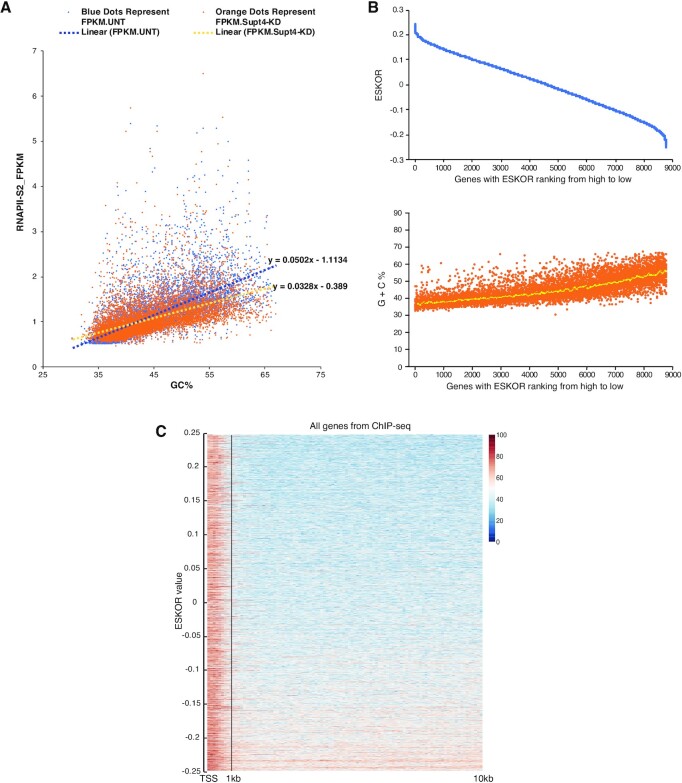
Effects of Supt4h reduction on DNA occupancy by elongating RNAPII are associated with G + C content of template. (**A**) DNA occupancy by elongating RNAPII was quantified by FPKM using ChIP-seq RNAPII-S2 data. RNAPII-S2 ChIP-seq FPKM (y-axis) of all 8779 genes with ChIP-seq FPKM > 0.5 were plotted with their G + C content (x-axis). Linear trendlines were added for Untreated (UNT) and Supt4h knockdown (Supt4-KD) conditions via Microsoft Excel. The equation of the trendline was displayed at the end of each trendline. (**B**) The effects of Supt4h reduction on DNA occupancy by elongating RNAPII were expressed as ESKOR values. Genes in which the calculated FPKM was >0.5 (8779 genes in total) were sorted according to their ESKOR value (y-axis) from high to low (upper panel). Using the same gene ranking (i.e. same x-axis as the upper panel), each gene's G + C content was plotted on the y-axis in the lower panel. The yellow dots represent the moving average of 100 neighboring genes (lower panel). (**C**) Heatmap analysis of G + C distribution in the first 10 kb of genes, sorted from high ESKOR to low ESKOR. Genes shorter than 10 kb were not included in the analysis. The first 10 kb of the gene body, including exons and introns, was scanned using a 100 bp sliding window starting at the TSS. The average G + C% for each sliding window was calculated along the gene for heatmap plotting. The G + C% is displayed by differing color as indicated.

As shown in Figure 1A, RNAPII-S2 ChIP-seq FPKM values differed according to the G + C content of the template segment being transcribed: on a genome-wide basis, template occupancy by RNAPII-S2 increased G + C content both in the presence and absence of DSIF complex. However, the slopes of the two Supt4h + and Supt4h- knockdown plots differed, and the effect of knockdown was greater on reads that were higher in G + C content. The linear trendline slope dropped 35% (from 0.0502 to 0.0328) after Supt4h was knocked down (Figure 1A).

The relationship between G + C content and effects of Supt4h knockdown on template occupancy by RNA polymerase was investigated further using a parameter we defined as ESKOR (Effect of Supt4h Knockdown On RNA polymerase occupancy). ESKOR values were defined as the log ratio of FPKMs obtained from Supt4h knockdown (KD) versus untreated (UNT) NPC cells: log_2_(RNAPII-S2 ChIP-seq_FPKM_KD/RNAPII-S2 ChIP-seq_FPKM_UNT). Altered template occupancy resulted in ESKOR values deviating from zero (Figure 1B). Overall, Supt4h production was reduced to ∼30% of the initial protein concentration (western blot quantification in Supplementary Figure S1A). RNA-seq showed Supt4h mRNA at 18% of its initial concentration (FPKM = 66.78 to 12.05 in Supplementary Table S2) resulted in a small percentage change in ESKOR (log_2_ differences of –0.25 to +0.24, indicating –16% to +18% shift from the baseline) (Figure 1B, upper panel) was observed under these conditions.

Global correlations between the effect of Supt4h knockdown on template occupancy (i.e. ESKOR) and template sequence were examined. When chromosomal genes were ranked from high to low according to ESKOR (Figure 1B, upper panel), and the percent of G + C nucleotides (36) for each gene was plotted (Figure 1B, lower panel and Supplementary Figure S1B), the ESKOR value was seen to vary with G + C content (Pearson correlation coefficient *r* = –0.78). Consistent with this correlation were the results of heatmap analysis of G + C content in 100bp sliding windows (Figure 1C) that extended in each gene from the TSS through the first 10 kb segment of genes.

To further understand genome sequence characteristics that affect ESKOR, we investigated additional correlations among the 8779 genes studied, (Supplementary Figure S2). Our analysis evaluated the frequency of the single nucleotides G and C in DNA regions where occupancy of the template by RNAPII is differentially affected by Supt4h. G and C percentages on the template strand showed high correlations with ESKOR, suggesting that dependency of RNAPII on Supt4h is affected similarly by these individual nucleotides. Notably, correlation between ESKOR and the dinucleotide sequence CpG, which commonly is a target for DNA methylation and alteration of nucleosome structure (37)—and which also has been reported to affect pausing by the RNA polymerase (38)—was no greater than correlation with GpC (Supplementary Figure S2C, D).

**Figure 2.**
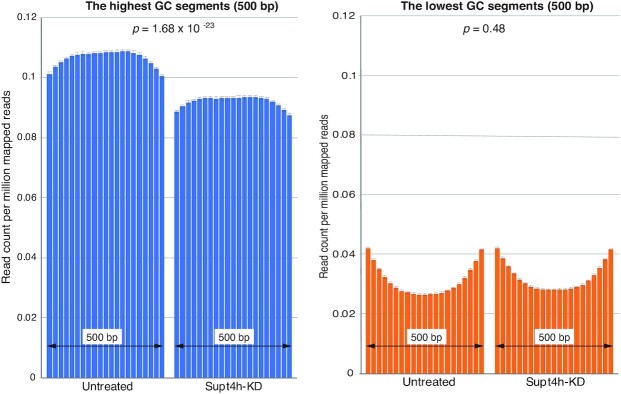
Comparison of the effects of Supt4h knockdown on high G + C and low G + C regions. Each of the 8779 genes identified in this analysis was computationally divided into segments of 500 bp in length, starting at a location 1 kb 3′ from the TSS. Segments having highest G + C and lowest G + C content of each gene were chosen, and read counts per million mapped reads were determined in the presence or absence of Supt4h knockdown. Read counts for these 500 bp segments were further divided into 25 bp bins and plotted (y-axis). The bars indicate the mean of the read count per million mapped reads, as determined by ChIP-seq analysis. The error bar represents the standard error of the mean.

To learn whether the effects of Supt4h reduction vary within genes according to the G + C content of the template segment being transcribed, we computationally divided each gene into 500 bp segments and determined RNAPII DNA occupancy for each gene's highest G + C segment and lowest G + C segment. The read counts from the highest G + C segment from each of the 8779 genes analyzed were combined and averaged (Figure 2). Read counts from the lowest G + C segments of the same 8779 genes were similarly combined and averaged (Figure 2). Analysis showed that even prior to Supt4h knockdown, the highest G + C segments had greater template occupancy by RNAPII-S2 than the lowest G + C segments—as reflected by a 3.4-fold greater average read count (Figure 2, untreated samples). Supt4h knockdown reduced RNAPII occupancy of the highest G + C segments by approximately 13.5% (from 0.106 to 0.092 average read count per million mapped reads, *P* = 1.68 × 10^−23^), while read counts from the lowest G + C segments were barely affected by Supt4h knockdown (*P* = 0.48) (Figure 2).

### Relationship between ESKOR and transcript length

**Figure 3.**
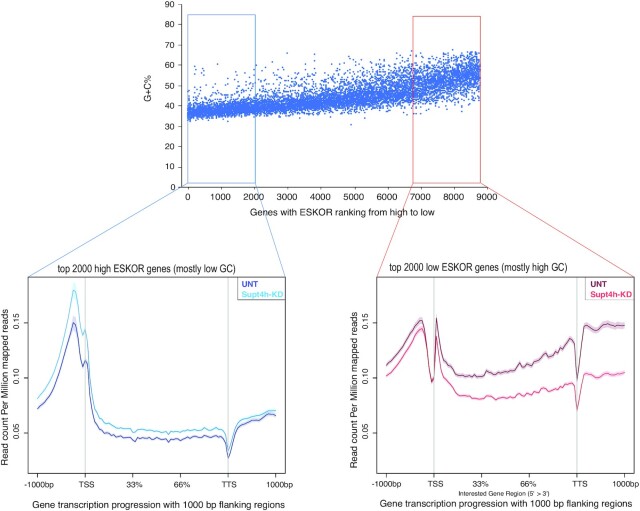
Comparison of the effects of Supt4h reduction on RNAPII-S2 occupancy of high ESKOR genes versus low ESKOR genes. 8779 genes having FPKMs >0.5 were sorted according to their ESKOR value (y-axis), and the 2000 highest ESKOR genes and 2000 lowest ESKOR genes were selected. For each of these genes, the gene body was divided, independently of gene length, into 100 bins. The normalized and aggregated read counts from the 100 bins for the gene bodies of each of the 2000 genes are shown for untreated cells and cells in which Supt4h has been knocked down in ngs.plot (62). Data for 1000 bp regions flanking each gene body are also shown to indicate read counts in promoter regions 5′ to transcription start sites and read counts for regions 3′ to transcription termination sites.

Transcript length and the location of a template sequence within genes also affected the extent of polymerase dependency on Supt4h to maintain its occupancy of the template. We determined this globally by examining the polarity of ESKOR values in 2000 genes showing the highest ESKOR values and 2000 genes showing the lowest ESKOR. ChIP-seq reads in each of these two groups were plotted across the whole gene body—from 1000 bp 5′ to the TSS to 1000 bp 3′ to the TTS (Figure 3). The results showed progressive reduction of RNAPII-S2 occupancy from TSS to TTS in genes showing low ESKOR values, but not in high ESKOR genes. However, RNA transcription was not substantially different (Supplementary Figure S3). As ESKOR values correlated inversely with G + C content (Figure 1), these data suggest that globally, continuing dissociation of RNAPII-S2 from high G + C templates occurs when DSIF clamping function is impaired, and consequently that RNAPII dependence on DSIF to prevent dissociation from G + C-rich template sequences increases with gene length. Screenshot examples of data from separate experiments for two high G + C content genes and two low G + C genes that illustrate progressive reduction of RNAPII-S2 occupancy from TSS to TTS are shown in Supplementary Figure S4.

**Figure 4.**
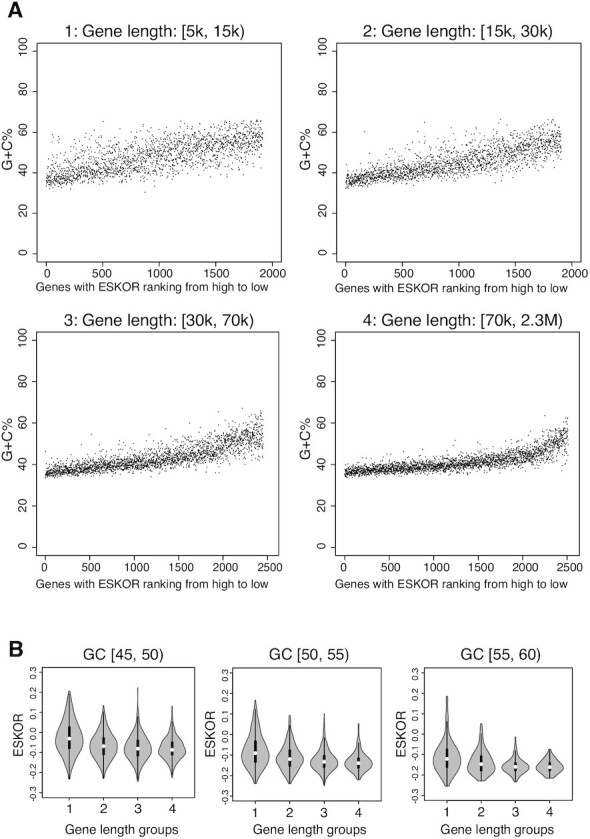
Association of gene length with ESKOR. (**A**) The 8779 genes were sorted into four groups according to gene length (5 kb ≤ group1 < 15 kb; 15 kb ≤ group 2 < 30 kb, 30 kb ≤ group 3 < 70 kb; 70 kb ≤ group 4 < 230 kb). Genes in each group were further sorted according ESKOR (high to low), and G + C% of individual genes was plotted as in Figure 1B. (**B**) Within each group of genes categorized by length, genes were grouped into three categories according to G + C content (45% ≤ G + C range 1 < 50%; 50% ≤ G + C range 2 < 55%, 55% ≤ G + C range 3 < 60%) and ESKOR values were compared. The ESKOR values (y-axis) from the four different length groups were plotted for the indicated G + C content range by violin plot. The white dot on the violin plot is the median. The black bar in the center of violin indicates the interquartile range, i.e. 25–75%.

Sorting genes into four groups according to their length (Figure 4A), we divided genes of each group according to their G + C content. Genes having a G + C content between 45% and 60% were assigned to one of three groups (45–50%, 50–55%, 55–60%) (Figure 4B). The ESKOR value distributions among genes having a similar G + C content but different lengths were compared. As seen in Figure 4, and consistent with results shown in Figure 3, this analysis showed greater effects of Supt4h reduction on longer genes than on shorter genes having a similar G + C content. Analogous results have been obtained by Fitz *et al.* (39), who investigated the relationship between Supt5h deficiency and gene length.

**Figure 5.**
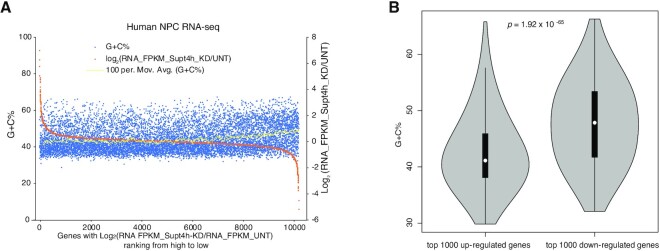
Association of G + C content with the effects of Supt4h reduction on gene expression. (**A**) Cell samples used for RNAPII-S2 ChIP-seq analysis were also used for RNA-seq assays. Gene expression changes in cells having Supt4h knockdown were sorted according to the extent of change by comparing their ratio Log_2_ (RNA_FPKM_KD/RNA_FPKM_UNT) (orange dots, right-hand y-axis). The G + C percentage for each gene (blue dots, left-hand y-axis) was plotted as shown in Figure 1B for ChIP-seq analysis. The yellow dots are the moving average G + C% of 100 neighboring genes. (**B**) G + C content from the most up-regulated 1000 genes and most down-regulated 1000 genes were analyzed by violin plot. The white dot on the violin plot is the median. The black bar in the center of the violin indicates the interquartile range, i.e. 25–75%.

RNA-seq analysis in NPCs showed overfall gene expression changes consistent with our ChIP-seq results for genes having different G + C content (Figure 5). RNA-seq data (GEO accession number GSE33497) obtained from mouse striatal cells (16) showed similar Supt4h-mediated downregulation of the expression of genes having high G + C content (Supplementary Figure S5).

### Effects of Supt5h knockdown on template occupancy by RNAPII

Knockdown of either Supt4h or Supt5h protein has been reported to be associated with concomitant reduction of the other (40), and evidence showing that depletion of Supt5h in murine embryonic fibroblasts leads to re-distribution of transcriptionally engaged RNAPII complexes within a narrow window near promoters has been published ((39); GEO accession number GSE106313). Further analysis of those data by us showed that the effects of Supt5h on RNAPII-S2 occupancy of the template also vary globally with the G + C content of the gene being transcribed (Supplementary Figure S6). These results support the conclusion that the ESKOR correlations we observed for Supt4h are attributable to the DSIF complex rather than some unrelated effect of Supt4h.

## DISCUSSION

The DSIF complexes formed by the binding of Spt4 to Spt5 in yeast, plants, and Archaea (41), or by interaction of their orthologs Supt4h and Supt5h in mammalian cells, perform functions essential to the synthesis of transcripts on DNA templates (4–6). X-ray diffraction and NMR studies indicate that by interacting with RNAPII, DSIF forms a clamp structure that closes a polymerase cleft cradling the DNA template—limiting dissociation of the enzyme from the template and facilitating enzyme processivity and transcript elongation (12,42,43), NusG, a prokaryotic orthologue of Spt5, carries out analogous functions in the absence of Spt4 (44,45). The observations reported here indicate that the clamping function of DSIF is utilized differentially on different segments of the genome, depending on the G + C content of the transcriptionally engaged template segment.

The experiments reported here were prompted by earlier observations showing that Supt4h or Supt5h knockdown can differentially affect alleles containing expanded nucleotide repeats (16). Our analysis of the genome-wide effects of Supt4h or Supt5h reduction on RNAPII-S2 occupancy in human neuron progenitor cells and mouse embryonic fibroblasts indicates that template occupancy by RNAPII is affected more prominently by DSIF during transcription of DNA regions that contain a higher proportion of G + C nucleotides.

RNAPII occupancy of DNA templates is known to be affected by pausing of the polymerase during transcript elongation (46,47) as well as by pausing in promoter-proximal gene regions (13,48). Our results support a model in which clamping of the polymerase onto templates by DSIF during transcript elongation reduces dissociation from the template, preventing premature termination of transcripts (49,50). Independently of DSIF function, pausing of RNAPII-S2 during its transit along DNA templates occurs at G + C nucleotide pairs at a higher frequency than at A + T nucleotide pairs (51); this effect has been attributed to the higher energy required to separate G–C bonds relative to A–T bonds (52). The data reported here indicate that template occupancy is also increased in template regions rich in G + C nucleotides in either the presence or absence of normal DSIF function and show also that reduction of DSIF function has a greater effect on polymerase occupancy of G + C-rich template sequences than on occupancy of A + T-rich template sequences. These findings support a model in which RNAPII-S2 relies on DSIF differentially to alter the *consequences* of polymerase pausing rather than the *frequency* of pausing.

As Supt4h and Supt5h are needed for normal functioning of mammalian cells, extensive knockdown can result in broad effects on cellular RNA synthesis (53), whereas partial knockdown is associated with limited changes in RNAPII-S2 template occupancy and RNA synthesis (Figures 1 and 5; see also (16,18,39)). As introns were included in our calculations of G + C content, and total gene length and G + C content are determined largely by sequences present in introns (54), intronic sequences, which are less conserved among species than exonic sequences (55) substantially affect the template occupancy we have reported.

Transcription through genes containing repeat expansions in Huntington's Disease (the *HTT* gene) (16–18), Amyotrophic Lateral Sclerosis (ALS) and Frontotemporal Dementia (FTD) (*C9orf72*) (20,21,56), and Spinocerebellar Ataxia type 36 (SCA36) (*NOP56*) (22) has been reported to be affected differentially by knockdown of Supt4h or Supt5h. In these diseases, expansion of pathogenic nucleotide repeats (CAG in Huntington's disease, GGGGCC in ALS/FTD; GGCCTG in SCA36) generates long template segments that have a G + C content between 66% and 100%. Expansion of repeats to yield long G + C-rich template segments is also associated with other genetic neurodegenerative disease genes, including Fragile X (CGG repeats), Myotonic Dystrophy type 2 (CCTG repeats), and Spinocerebellar Atrophy types 1 through 3 (CAG repeats) (57,58). Such long G + C-rich DNA segments are rare in non-mutated human genes. In Huntington's Disease, interruption of the continuity of CAG repeats by trinucleotide repeats (CAA) that encode the same amino acid residue as CAG but have a G + C content of only 33% can affect the age of onset of clinical symptoms (59–61). We suggest that the influence of G + C content on DSIF-mediated template occupancy by RNAPII may have phenotypically important consequences in a broad range of nucleotide repeat diseases.

In addition to performing ChIP-seq in NPC samples, we re-analyzed previously published ChIP-seq data obtained from mouse embryonic fibroblasts in which Supt5h was absent (39). Notwithstanding a species difference (human versus mouse), a cell type difference (NPCs versus fibroblasts), and a difference in the DSIF component altered (Supt4h versus Supt5h), our analysis of the Fitz et al. data showed that dependence on DSIF for RNAPII occupancy of template sequences was similarly affected by G + C content.

## DATA AVAILABILITY

The sequencing data generated in this study have been submitted to the Gene Expression Omnibus (GEO; https://www.ncbi.nlm.nih.gov/geo/) under accession number GSE169466.

## Supplementary Material

lqac054_Supplemental_File

## Notes

Present address: Ning Deng, BridGene Biosciences, San Jose, CA 95134, USA.

Present address: Yue Zhang, Earli Inc., South San Francisco, CA 94080, USA.

Present address: Zhihai Ma, Chapter Diagnostics Inc., Menlo Park, CA 94025, USA.
