# Supplementary material for: DSIF modulates RNA polymerase II occupancy according to template G + C content
Source: NAR Genom Bioinform. 2022 Jul 27;4(3):lqac054. doi: 10.1093/nargab/lqac054 (PMC9326580; doi:10.1093/nargab/lqac054)
Supplement: lqac054_Supplemental_File [file lqac054_supplemental_file.pdf]

Supplementary Data For:

DSIF modulates RNA polymerase II occupancy according to template G+C content

Ning Deng[1,2 †], Yue Zhang[1,3 †], Zhihai Ma,[1,4, †], Richard Lin[1], Tzu-Hao Cheng[5], Hua Tang[1], Michael P. Snyder[1] & Stanley N. Cohen[1,*]

[1]Department of Genetics, Stanford University School of Medicine, Stanford, CA, 94305, USA,

[5]Institute of Biochemistry and Molecular Biology, National Yang Ming Chiao Tung University, Taipei, 112, Taiwan

[2]Current address: BridGene Biosciences, San Jose, CA 95134, USA, [3]Current address: Earli Inc., South San Francisco, CA 94080, USA, [4]Current address: Chapter Diagnostics Inc., Menlo Park, CA, 94025, USA

* To whom correspondence should be addressed: Tel: +1 (650) 723-5315; Fax: +1 (650) 725-1536; Email: sncohen@stanford.edu

[†]The authors wish it to be known that, in their opinion, the first three authors should be regarded as Joint First Authors.

Supplementary Table S1: Statistical Information for Human NPC ChIP-seq Data.

Condition	Cell Lines	Total Raw Reads	Reads After Removing Adaptor	Mapping Rate
Untreated RNAP2S2	GM23225 NPC	367549175	367543258	97.55%
Supt4h-KD RNAP2S2	GM23225 NPC	388916388	388910219	92.90%

Supplementary Table S2: Statistical Information for Human NPC RNA-seq.

Condition	Cell Lines	Total Raw Reads	Mapping Rate	SUPT4H Expression (FPKM)
Untreated	GM23225 NPC	55850420	88.58%	66.78
Supt4h-KD	GM23225 NPC	61416842	86.15%	12.05

Supplementary Table S3: Statistical Information for Mouse Striatum Cell Line RNA-seq Data.

Condition	Species	SRA Accession Number	Total Raw Reads	Mapping Rate	SUPT4h Expression (FPKM)
Untreated	Mouse	SRR363972	86014617	74.46%	156.28
Supt4h-KD	Mouse	SRR363973	90625898	74.72%	74.11

A

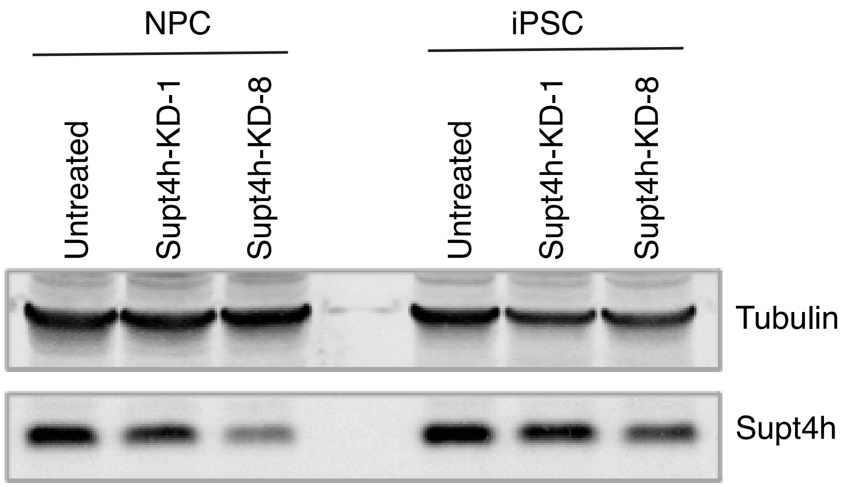

B

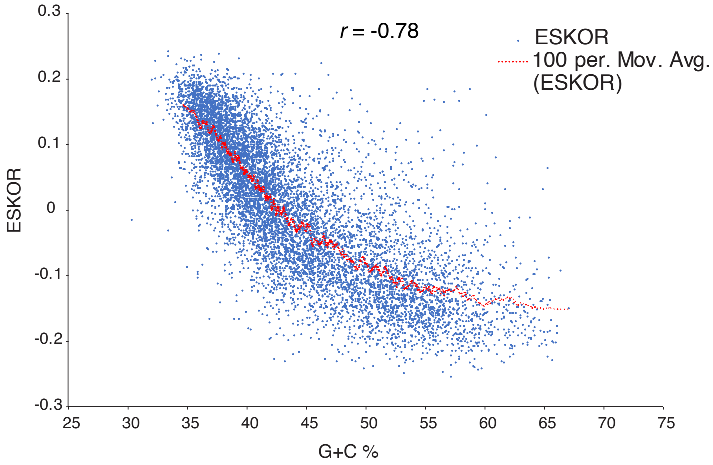

Supplementary Figure S1. Effects of Supt4h knockdown by shRNA. (**A**) Protein isolated from NPC and iPSCs were measured by Western blotting, respectively, in untreated cells or after stable knockdown of Supt4h by lentivirus carrying Supt4h shRNA. According to quantification of Supt4h band in the Western Blot with Li-Cor Image Studio software, Supt4h in NPC clone 8 was 27% left compared to untreated sample. Clone 8 in NPC was used for further ChIP-seq and

RNA-seq. (**B**) ESKOR (y-axis) was plotted against G+C% (x-axis). The red dots represent the ESKOR moving average for 100 genes.

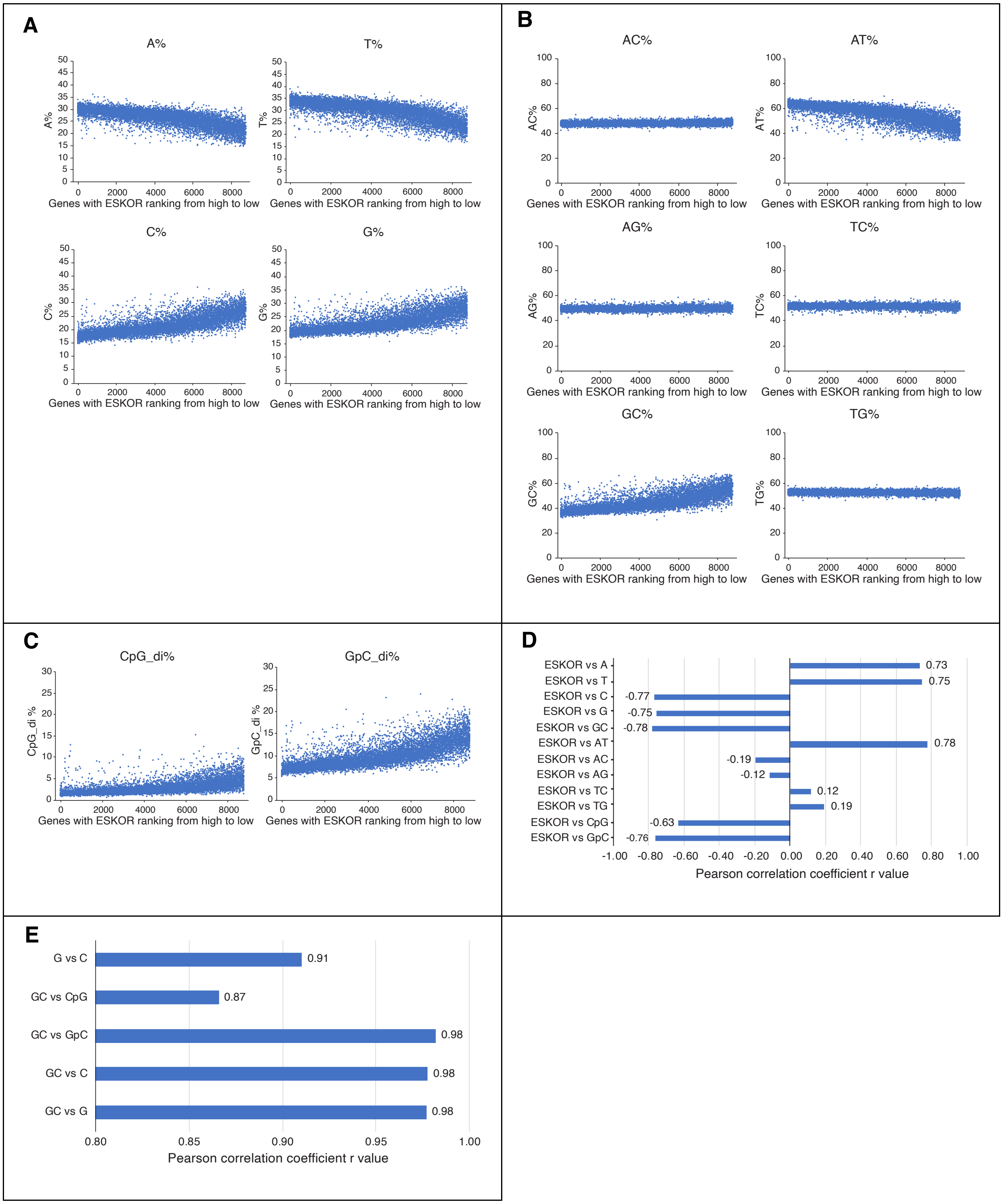
A
A%
T%
C%
G%
A%
T%
C%
G%
Genes with ESKOR ranking from high to low
B
AC%
AT%
AG%
TC%
GC%
TG%
Genes with ESKOR ranking from high to low
C
CpG_di%
GpC_di%
CpG_di %
GpC_di %
Genes with ESKOR ranking from high to low
D
ESKOR vs A 0.73
ESKOR vs T 0.75
ESKOR vs C -0.77
ESKOR vs G -0.75
ESKOR vs GC -0.78
ESKOR vs AT 0.78
ESKOR vs AC -0.19
ESKOR vs AG -0.12
ESKOR vs TC 0.12
ESKOR vs TG 0.19
ESKOR vs CpG -0.63
ESKOR vs GpC -0.76
Pearson correlation coefficient r value
E
G vs C 0.91
GC vs CpG 0.87
GC vs GpC 0.98
GC vs C 0.98
GC vs G 0.98
Pearson correlation coefficient r value

Supplementary Figure S2. Relationship of ESKOR to nucleotide content in 8779 genes. (**A,B**) Correlation of ESKOR with genome incidence of single nucleotides (**A**) or double (**B**) nucleotide combinations. Genes were ranked according to ESKOR. Blue dots represent each gene's single or double nucleotide content in the gene body as shown on the y axis. (**C**) Plotting of ESKOR for CpG and GpC di-nucleotide combinations as in (**A**). (**D**) Pearson correlation coefficient to quantify ESKOR vs. indicated nucleotide composition for all 8779 genes is shown. (**E**) Pearson correlation coefficient between indicated nucleotides or nucleotide combinations.

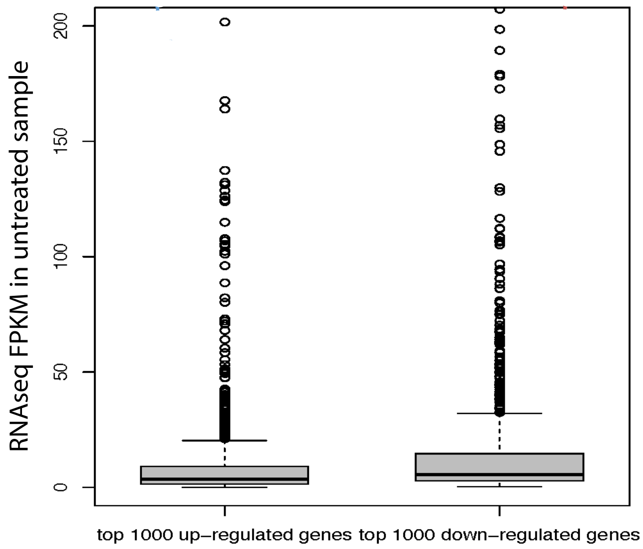

Supplementary Figure S3. No significant expression level difference was observed between the top 1000 up-regulated genes (high ESKOR genes, mostly low GC) and top 1000 down-regulated genes (low ESKOR genes, mostly high in GC) in Figure 3. We analyzed the 1000 highest and 1000 lowest ESKOR genes' expression in untreated sample by RNA-seq.

Low ESKOR gene examples	High ESKOR genes examples

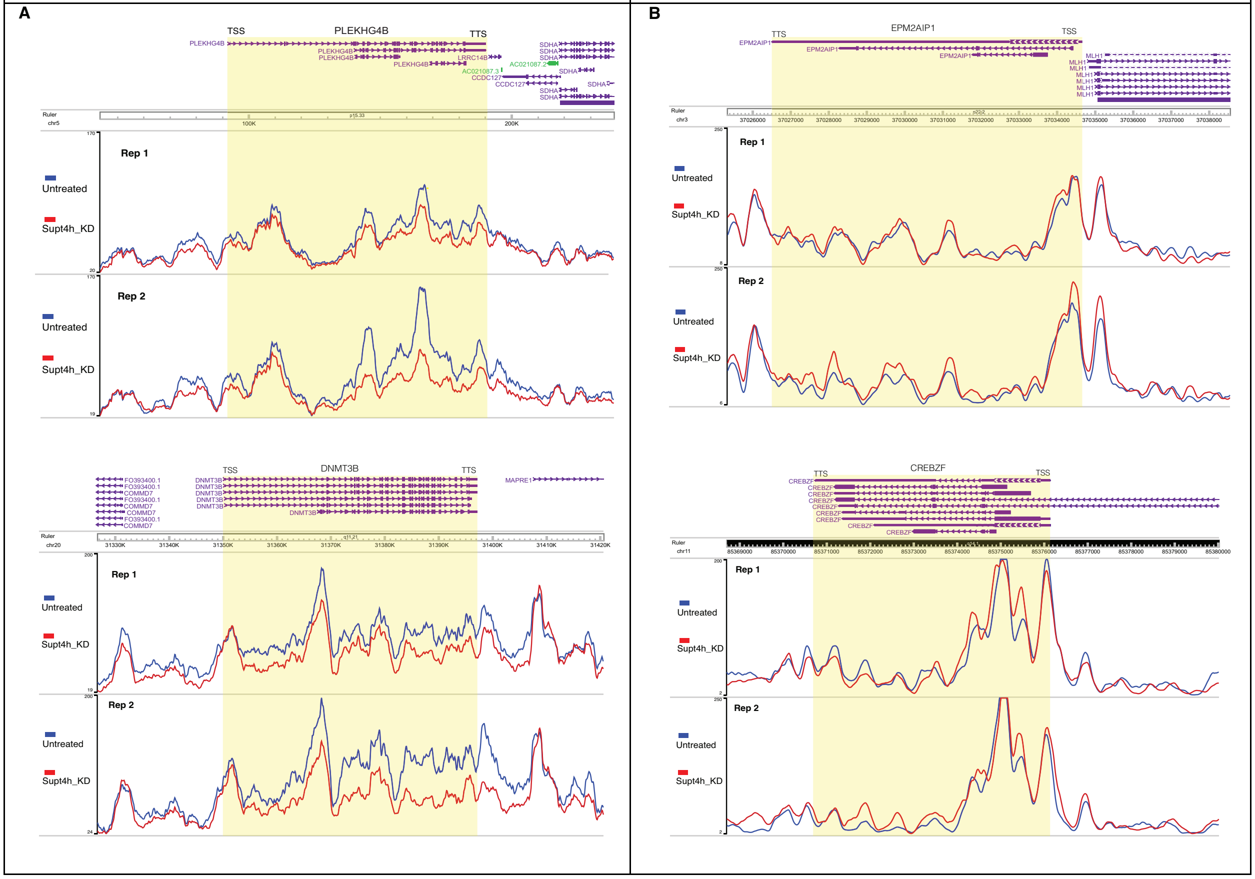

Supplementary Figure S4. Screenshot examples of RNAPII-S2 ChIP-seq signal from **A.** Low ESKOR genes (high GC) and **B**. high ESKOR genes (low GC). RNAPII-S2 ChIP-seq reads are displayed in WashU Epigenome Browser format. The blue (untreated sample) and red (Supt4h-KD sample) peaks/lines indicate RNAPII-S2 occupancy in two replicates as assayed by ChIP-seq analysis. Yellow shading indicates the boundaries of the gene segment extending from TSS to TTS.

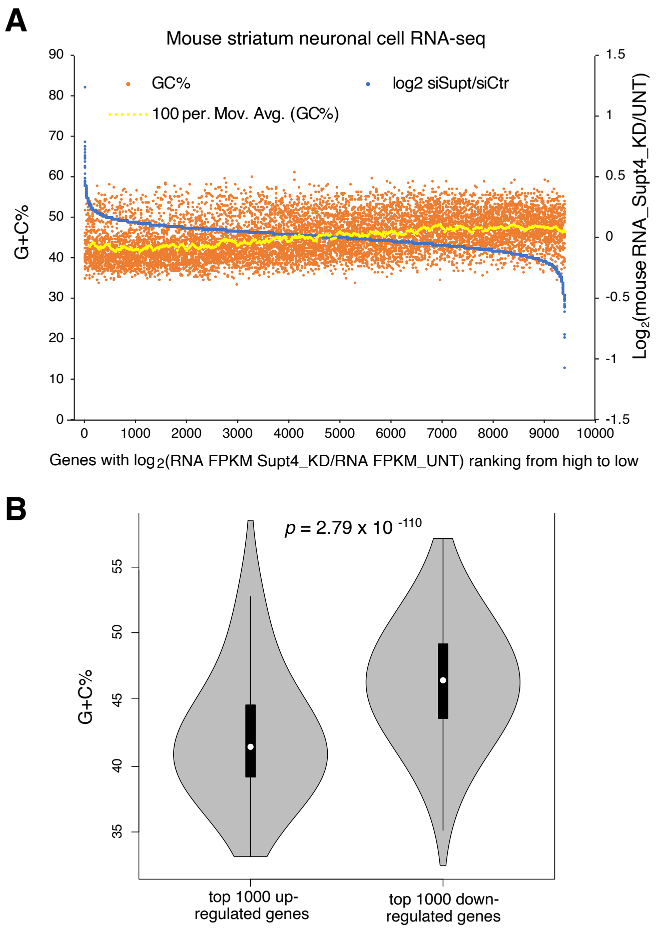

Supplementary Figure S5. Correlation of G+ C content with the effects of Supt4h reduction on gene expression in mouse striatum cells. We re-analyzed our previously published RNA-seq data (16) obtained from a non-disease mouse striatum neuronal cell line (Hdh $^{Q7/Q7}$) before and after Supt4h knockdown by transient transfection of siRNA targeting Supt4h using procedures described in Figure 5.

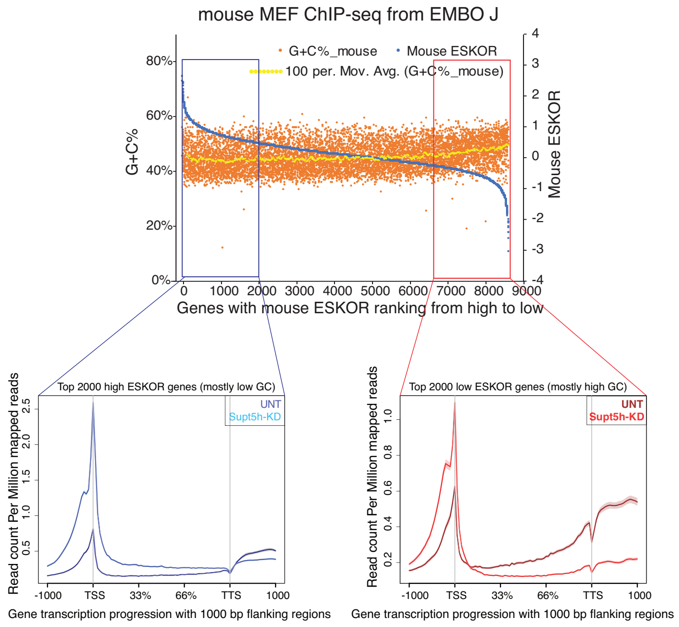

Supplementary Figure S6. Analysis of ChIP-seq data from Supt5h depletion mouse embryonic fibroblast cells. ChIP-seq data showing the effect of Supt5h depletion on template occupancy by RNAPII-S2 were published previously (38). G+C content and ESKOR for individual genes were determined as described for Figure 1A. Analyses were performed as in Figures 1A and 3.

A

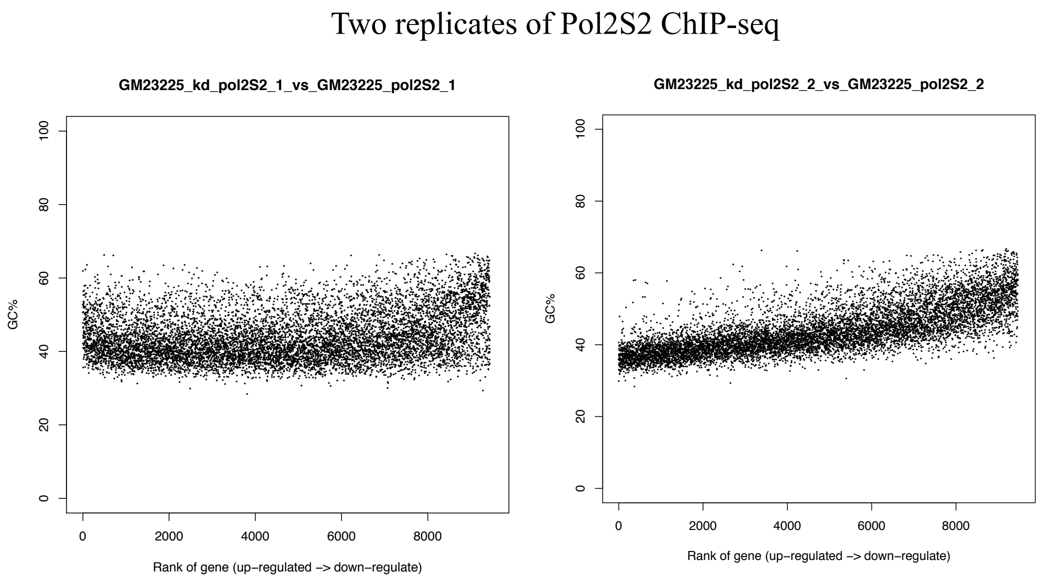

B

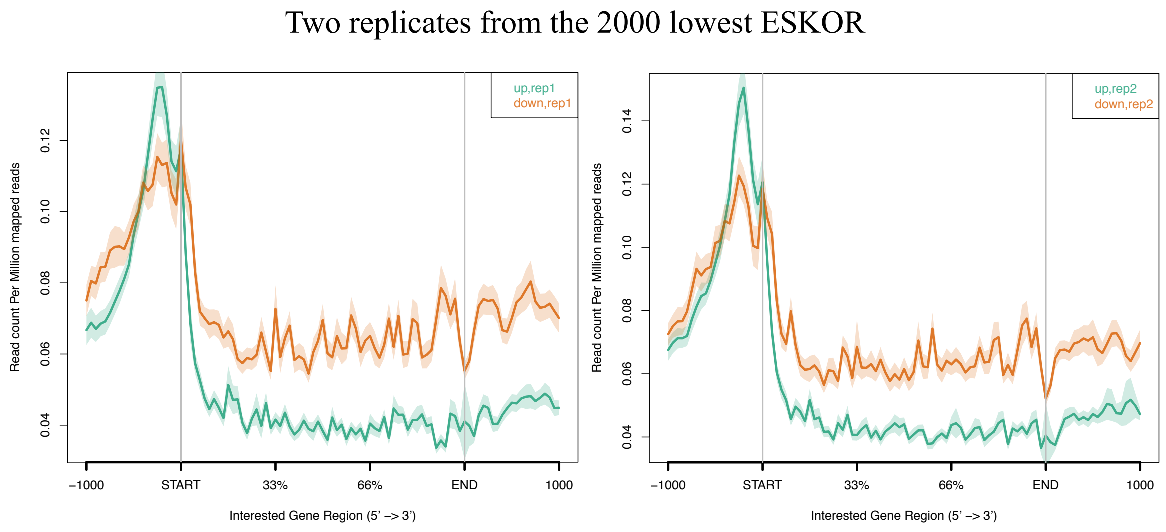

Supplementary Figure S7. ChIP-seq results for two replicates of samples. (**A**) The two replicates of ChIP-seq were analyzed as described in Figure 1A. (**B**) The 2000 lowest ESKOR genes from the two replicates were analyzed as described in Figure 3.
